# Optimizing emergency shutdown system inspection, testing, and maintenance through the tool design and validation

**DOI:** 10.1038/s41598-025-20964-0

**Published:** 2025-10-22

**Authors:** Amin Babaei-Pouya, Seyyed Bagher Mortazavi, Omran Ahmadi

**Affiliations:** Department of Occupational Health and Safety Engineering, Faculty of Medical Sciences, Tarbiat Modares University, Box: 331-14115, Tehran, Iran

**Keywords:** Inspection testing and maintenance (ITM), Emergency shutdown (ESD), Asset integrity management (AIM), Petrochemical, Occupational health, Mechanical engineering

## Abstract

Emergency Shutdown (ESD) systems serve as reliable control mechanisms within the petrochemical industry. These systems enhance safety by automatically shutting down processes during emergencies, mitigating hazards. The effectiveness of ESD systems is closely linked to robust practices in inspection, testing, and maintenance (ITM). This study aims to optimize the ITM of ESD systems through an asset integrity management (AIM) approach in the petrochemical industry located in the Assaluyeh region of Iran, focusing on the analysis of individual and organizational factors as well as the design and validation of a specialized assessment tool. The research follows a structured methodology. The first step involves identifying, screening, and validating the individual and organizational factors that influence the effectiveness of ITM for ESD systems. This is achieved through a literature review and expert opinions gathered via the Fuzzy Delphi method. The second step entails the development of tool items based on the literature review and expert feedback, along with verifying reliability and validity. By concentrating on aspects such as planning, supervision and support, documentation, communication, training, safety, and the integration of new technologies and artificial intelligence, organizations can significantly enhance their ITM practices. The tool designed for assessing individual and organizational factors in the ITM of ESD systems demonstrated strong validity and reliability. The validation of this tool underscores the importance of having accurate instruments to evaluate current conditions and future requirements, ultimately leading to the optimization of ESD systems.

## Introduction

Asset Integrity Management (AIM) integrates appropriate equipment, reliable individual performance, and effective management systems to enhance safety and efficiency in operations^1^. The benefits of AIM include improved equipment reliability and availability, reduced asset failure rates, and lower operating costs^2^. A key focus of AIM is the development and implementation of inspection, testing, and maintenance (ITM) practices^3^. Strengthening AIM involves analyzing the contributions of both individual and organizational factors, as ITM serves as the core of AIM^4^. The primary purpose of ITM is to identify and implement tasks that effectively control process risks^5^. ITM program activities often span multiple departments within an organization^6^. Emergency Shutdown (ESD) systems, crucial in the oil, gas, and petrochemical industries, are designed to automatically shut down processes during emergencies to enhance safety^7^. The main objectives of an ESD system are to protect personnel, safeguard the plant and environment, and minimize production downtime and damage to assets when processes exceed control limits^8^. ESD systems process incoming signals and activate output commands based on a predefined cause-and-effect diagram for the facility^9^. Key components of an ESD system include sensors and detectors, a logic processing unit, emergency shut-off valves, emergency evacuation systems, control panels, and alarms and indicators^10^. These systems must maintain a high level of safety, with their effectiveness heavily reliant on robust ITM practices^11^. Optimizing ITM is essential for the reliability of ESD systems, posing a challenge for organizations to ensure that ITM practices are effective, consistent, and compliant with industry standards ^12^. Numerous catastrophic failures have been linked to inadequate AIM^13^. Four asset-related factors (construction, testing, inspection, and maintenance) account for over half (54%) of accidents. Moreover, individual factors directly (23%) and indirectly (11%) contribute to accidents and other technical failures (15%)^14^. A study by Ramasamy^15^ emphasizes the importance of AIM as a key contributor to accidents, while research by Pan^16^ identifies leakage, fire, and explosion as the most frequent chemical accidents, highlighting the critical role of ESD systems in their control. Additional studies by XIE, Schonbeck, Hauge, and Deng have examined safety instrumented systems, which encompass various components in process industries^17–20^. In this study, we focused exclusively on the ESD system for a detailed examination. As the petrochemical industry evolves, maintaining the effectiveness of ESD systems through structured ITM practices is crucial for ensuring safety and business continuity. Previous research has been conducted on various aspects of asset integrity: Schmitz investigated the mechanical integrity of alarms, Sattari explored the strengthening of asset integrity, and Chin focused on optimizing asset maintenance^1,21,22^. For asset integrity, it is essential to specifically consider ITM. Zhu analyzed the workflow and identified gaps in the ESD system, emphasizing the need for a thorough investigation of the individual and organizational factors influencing this system^7^. Additionally, studies by Duma, Schönbeck, and Pan highlight the significance of individual and organizational factors in process safety management^16,18,23^. Various studies have focused on designing, developing, and validating tools to assess safety culture and maintenance practices in the petrochemical industry. Seyed et al. proposed an integrated maintenance model with four main components: human, management, knowledge, and equipment ^24^. Rad et al. and Jafari et al. developed and validated safety culture questionnaires tailored for the petrochemical industry, emphasizing the importance of reliability and validity in such tools ^25,26^. Jafari et al. identified ten key dimensions of safety climate, including management commitment and worker empowerment ^26^. Vianello et al. highlighted the need for effective inspection and maintenance planning tools to manage operational risks in complex chemical facilities^27^. Collectively, these studies underscore the importance of tailored assessment tools and models to improve safety and maintenance practices in the petrochemical sector, focusing on questionnaire design, validation, and the integration of multiple factors affecting safety and maintenance performance.

ESD systems, as critical safety components in petrochemical facilities, play a vital role in preventing major incidents, mitigating the consequences of process accidents, and protecting personnel and the environment. Given the technical complexity of ESD systems and their critical need for error-free performance in emergencies, improving the efficiency of inspection, testing, and preventive maintenance processes is essential to ensure their reliability and availability. AIM provides a comprehensive framework that goes beyond isolated measures, considering the interplay between technical assets, human factors, processes, and organizational structures. In this study, to optimize ITM of ESD systems, individual factors and organizational factors are analyzed to identify variables affecting ITM performance. Additionally, a measurement tool based on quantitative and qualitative indicators is designed and validated to assess the effectiveness of testing and maintenance programs. The expected results could facilitate risk-based decision-making, reduce unexpected failures, and enhance the sustained performance of ESD systems in the petrochemical industry. This study aimed to conduct a comprehensive analysis of the individual and organizational factors influencing the ITM of ESD systems within the context of AIM. Furthermore, it seeks to design and validate a tool that effectively assesses these factors, providing valuable insights for practitioners in the field. By emphasizing the importance of a systematic approach to ITM and AIM, this research supports the development of best practices that can enhance safety and operational performance in the petrochemical industry. Ultimately, this research contributes to the existing literature by demonstrating that optimizing ITM practices through a focus on individual competencies and organizational support can lead to improved system reliability in the industry. This study specifically aimed to optimize the ITM of ESD systems using an AIM approach by analyzing individual and organizational factors and designing and validating the assessment tool.

## Materials and methods

### Study design

**Fig. 1:**
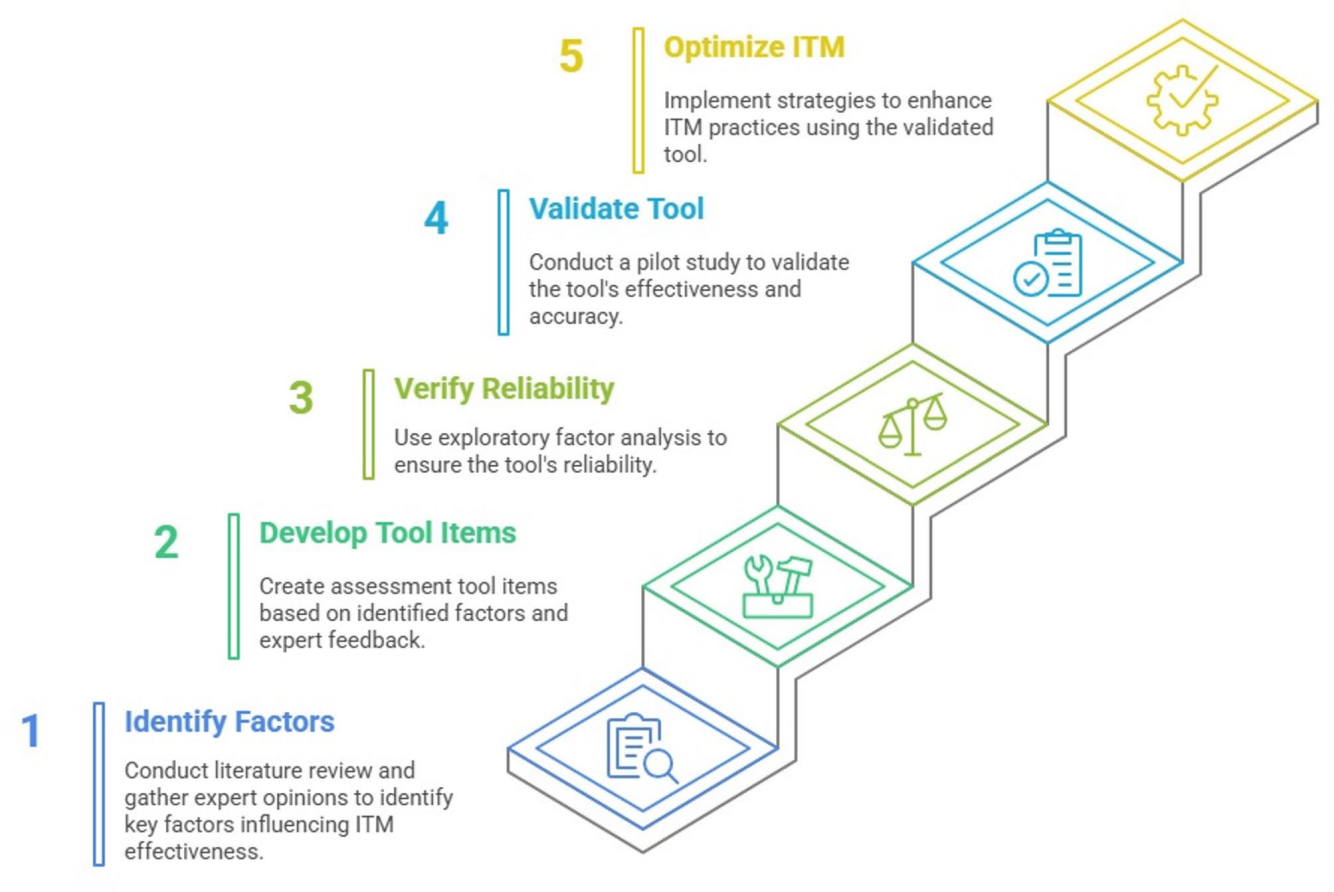
Research steps.

This study employs an applied, descriptive, and cross-sectional design. The primary objective is to identify, screen, and confirm individual and organizational factors affecting the ITM of Emergency Shutdown (ESD) systems, utilizing an AIM approach within the Iranian petrochemical industry. The research process consists of the following stages: The first stage involves identifying, screening, and confirming the individual and organizational factors that influence the ITM of ESD systems. This is achieved through a literature review and expert consultations, utilizing the fuzzy Delphi method. The second stage focuses on compiling the tool items by reviewing the literature and gathering expert opinions. This stage also includes verifying the tool’s reliability and validity through exploratory factor analysis using SPSS16, followed by confirmatory factor analysis via a pilot study with SmartPLS4 (Fig. 1).

### Review study

The identification of individual and organizational factors relevant to the ITM program of the ESD system was conducted through a review study. In this study, relevant articles were identified through a systematic search of standard keywords in reputable databases covering the period from 2020 to 2025. A systematic search was executed using keywords such as “petrochemical,” “chemical process,” “ESD system,” “safety instrument systems,” and “inspection, testing, and maintenance.” A tailored search strategy was employed for each database, utilizing the OR and AND operators. Inclusion criteria required that articles contain the terms “petrochemical,” “ Emergency Shutdown system,” and “maintenance” in the title, and “petrochemical,” “chemical process,” “safety instrument systems,” “ Emergency Shutdown system,” and “ inspection, testing, and maintenance” in the abstract or keywords. The PRISMA protocol guided the screening and evaluation of articles, while studies such as letters to the editor and theses were excluded from consideration. Duplicate and irrelevant articles were eliminated, and the titles and abstracts were reviewed. In the final stage, the full texts of the selected articles were evaluated by the researcher. Ultimately, 32 articles were finalized and included in the study.

### Study experts

The Asaluyeh Special Economic Zone hosts numerous gas complexes, refineries, and large petrochemical plants, making it one of Iran’s most significant petrochemical industry hubs. The complex process equipment and dense critical infrastructure, including pipelines, sensitive process units, and safety systems, make Asaluyeh an ideal environment for studying issues related to inspection, testing, and preventive maintenance. Additionally, the operational nature and safety sensitivities of these complexes necessitate asset integrity management approaches and risk-based solutions, linking study outcomes to practical applications and industrial policymaking.

For this study, 15 experts, including operations engineers, maintenance personnel, safety specialists, and technical managers from the Asaluyeh Special Economic Zone in southern Iran, were selected. The selection criteria included practical availability, willingness to cooperate, and a minimum of five years of specialized experience in planning and executing inspection, testing, and maintenance of ESD systems and related assets. The selection of this expert sample, due to their high focus and functional diversity in the petrochemical industry, provides access to a wide range of practical experiences, enhancing the content validity of the designed tool in real industrial contexts. Furthermore, the experts’ experiences in addressing operational challenges, constraints, and risk mitigation strategies contribute to identifying individual and organizational factors that affect the effectiveness of ITPM, ultimately improving the generalizability of the results to similar industrial contexts.

### Implementation of Fuzzy Delphi

The Fuzzy Delphi questionnaire was created by reviewing literature on individual and organizational factors that influence the ITM of the ESD system. This questionnaire was distributed to experts to identify, screen, and validate these factors. To convey the significance of each factor, we utilized linguistic variables and triangular fuzzy numbers: very low (0, 0, 0.25), low (0, 0.25, 0.5), medium (0.25, 0.5, 0.75), high (0.5, 0.75, 1), and very high (0.75, 1, 1). We calculated the triangular fuzzy values based on the experts’ opinions (refer to Formulas 1–5)^28^. To determine the average opinion of the 15 respondents, we computed their fuzzy average. In this context, index i denotes the expert, while index j refers to the decision-making index. We then derived the defuzzified value of the average fuzzy number. Factor screening involved comparing each factor’s acquired value against a threshold of 0.7. A factor was approved and finalized when the difference between the average values of two consecutive rounds of the Fuzzy Delphi process was less than 0.1^29^.

$${\widetilde{\tau }}_{\text{ij}}=\left({\text{a}}_{\text{ij}},{\text{b}}_{\text{ij}},{\text{c}}_{\text{ij}}\right),\text{ i}=\text{1,2},\dots ,\text{n j}=\text{1,2},\dots ,\text{m}$$

$${a}_{j}=\sum \frac{{a}_{ij}}{n}$$

$${b}_{j}=\sum \frac{{b}_{ij}}{n}$$

$${c}_{j}=\sum \frac{{c}_{ij}}{n}$$

$$Crisp= \frac{a+b+c}{3}$$

### Development of the initial instrument

Following the identification, screening, and validation of effective factors, the initial design of the instrument items was developed through a literature review. To assess the face validity of the instrument, we incorporated corrective comments from experts regarding the development of the items and factors. As a result, an initial instrument consisting of 43 questions was created.

### Pilot study

The pilot study was conducted in two phases: exploratory factor analysis and confirmatory factor analysis. This study aimed to validate the instrument within the petrochemical industry located in the Assaluyeh region, in coordination with the National Iranian Petrochemical Company. The pilot study was carried out both in person and online in January 2025. Data collection for the individual and organizational factor analysis was conducted in person, while the design and validation of the instrument took place online. The instrument was created using an online tool, and the link was shared with study participants via social networks and SMS, ensuring privacy. The statistical population included employees from planning, operations, supervision, and support units involved in the ITM of the ESD system. The tool was completed and validated using a convenience sampling method.

### Validity and reliability of the tool

To assess the content validity of the tool, expert judgment was utilized. The content validity ratio index (less than 0.49) was used to confirm the necessity of the items, while the content validity index (less than 0.79) was employed to ensure their relevance. The reliability of the tool was evaluated using the split-half method and Cronbach’s alpha coefficient (greater than 0.7). The final tool, consisting of 36 questions, was approved^30,31^.

### Exploratory factor analysis

The Kaiser-Mayer-Olkin (KMO) test was conducted to evaluate sample adequacy, with a value close to one (at least 0.6) indicating suitability for factor analysis. If Bartlett’s test significance level was less than 5%, it suggested a relationship between the variables. Based on the total variance explained, a series of factors were identified, with each factor having a total eigenvalue greater than 1 being selected. The Scree Plot chart illustrates the eigenvalue distribution, highlighting important factors with eigenvalues exceeding 1. The final clustering of the rotational component matrix was performed to identify the main factors, placing items with high correlations alongside their respective factors. The items were categorized based on which factors they measured, and the identified factors were named according to the nature of the items. After this stage, the main structure of the tool was established^32–34^.

### Confirmatory factor analysis

To evaluate the measurement model, we reported factor loadings, Cronbach’s alpha coefficient (greater than 0.7), composite reliability (CR) coefficient (greater than 0.7), Average Variance Extracted (AVE) (greater than 0.5), Fornell & Larcker criteria (greater than 0.6), Cross-Loadings, and Heterotrait-Monotrait Ratio (HTMT) (less than 0.9). To assess the model fit, the Standardized Root Mean Square Residual (SRMR) index (less than 0.08) was reported^35–37^.

### Ethical issues

All procedures were conducted according to the Declaration of Helsinki. The Ethics Committee of Tarbiat Modares University, Iran, reviewed and approved this study (Ethics Approval Code: IR.MODARES.REC.1403.054). Written informed consent was obtained from all participants prior to their involvement. Approval to carry out the research was granted by the committee, and participant confidentiality was strictly maintained in line with ethical standards throughout the study.

## Results

The review included a total of 32 articles (Table 1). From these articles, twenty factors were identified, including: knowledge, training, motivation, compliance, adaptability, job requirements, job control, job support, reward, fairness, participation, communication, planning, monitoring, standards, instructions and procedures, documentation, physical asset management, hazard identification and risk management, failure management, equipment management, and process safety critical tasks.

**Table1:** Individual and organizational ITM Factors.

Authors	Factors
Zhu et al.^8^	Technical, operational
AlHamouri et al.^38^	workface planning
Ali et al.^39^	false signal
Al-Douri et al.^40^	Root cause analysis and availability modeling
Deng and Wang^20^	Misoperation monitoring and early warning during
Zhu et al.^7^	Data-driven failure analysis, Evaluation of detectability from a failure progression perspective
Abdali et al.^41^	Improving responses to early warning signals, safety systems, equipment, and operating procedures
Hainen et al.^42^	Response plans, maintenance plans
Zuo et al.^43^	Reliability Study of Parameter Uncertainty, Time-Varying Failure Rates
Ismail et al.^44^	Implementation of an automation system-based model, managing imperfect maintenance actions
Zhen et al.^45^	Optimization of maintenance intervals, integrating risk and cost for safety–critical barriers
Behie et al.^46^	Process safety management systems
Liu et al.^47^	Modeling and performance analysis, Stochastic Petri nets
Adamyan et al.^48^	Development of technological measures, safety of production facilities
Zhu et al.^49^	Critical project planning and spare parts inventory management
Yin et al.^50^	Mechanization level assessment and technology identification
Han et al.^51^	Multi-objective optimization for maintenance, safety–critical equipment, integrating dynamic risk and maintenance cost
Di-Sarno et al.^52^	Risk assessment with ageing
Gonyora et al.^53^	Procedure implementation, communication accuracy, communication satisfaction, work permit system, competency level, and risk management
Oropallo et al.^54^	decision support system to assess the operational safety and economic benefits, risk-based inspection implementation strategies
Bouabid et al.^55^	Three fundamental pillars— environmental, economic, and social—underpin emerging trends in maintenance development. Maintenance management
Aghaee et al.^56^	fuzzy hybrid multi-criteria decision-making approach
Ekpe et al.^57^	Risk Perception
Abdul Jawwad et al.^58^	Analytical hierarchy process (AHP)
Mollo et al.^59^	Technology Acceptance Model
Ramos et al.^60^	human reliability analysis, Phoenix-PRO qualitative framework
Hosseini et al.^61^	Management, human resources, equipment, and knowledge
Shan et al.^62^	Mechanization and an assessment method
Shan et al.^63^	An extended fuzzy PROMETHEE II approach
De Vries et al.^64^	Knowledge and motivation. Availability of equipment, availability of spare parts
Chin et al.^22^	Corrective maintenance, time-based maintenance, risk-based maintenance, condition-based maintenance, and opportunistic maintenance
Zhang et al.^65^	Two-phase imperfect inspections

**Fig. 2:**
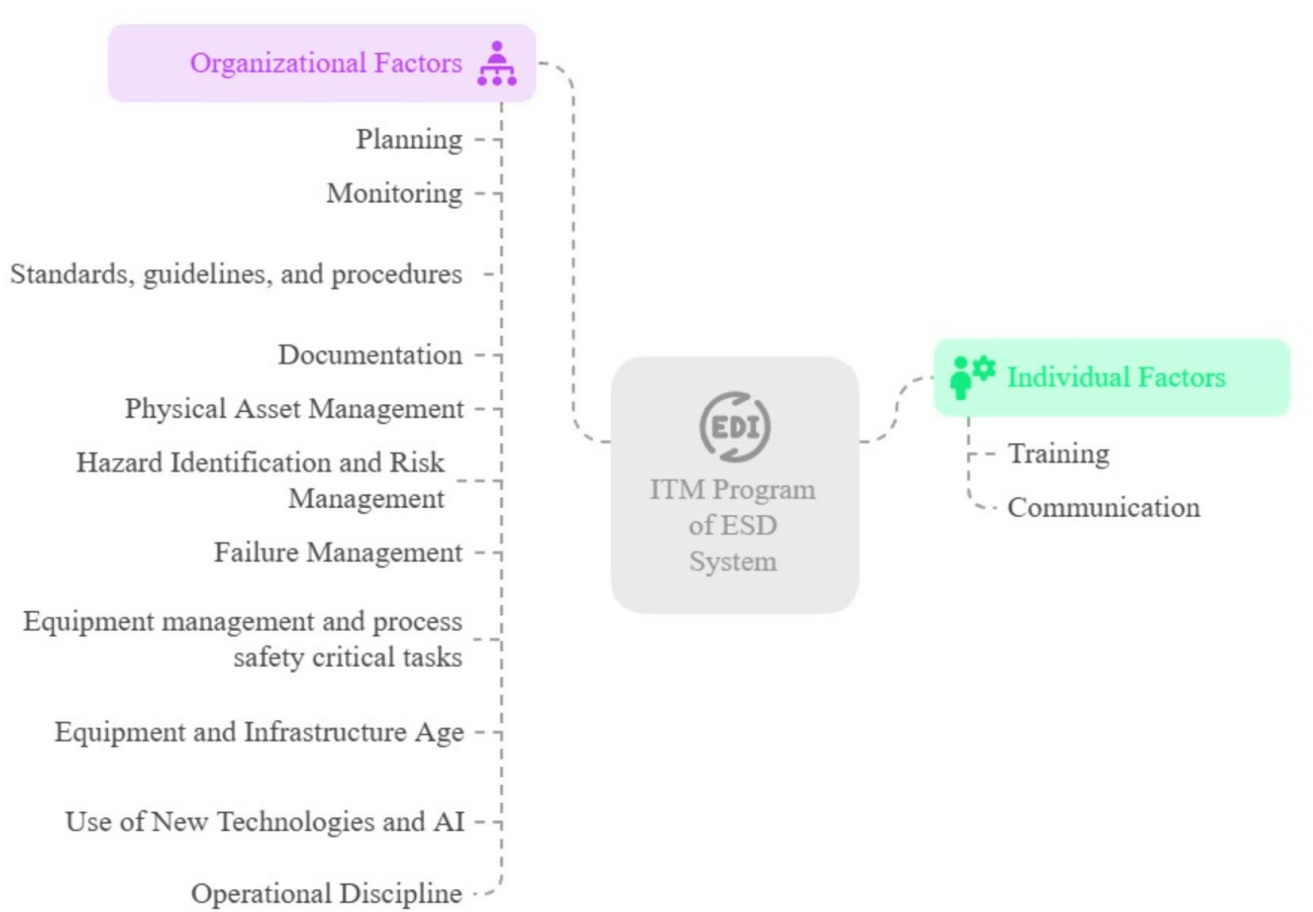
Factors optimizing the ITM of ESD Systems.

Individual and organizational factors influencing the ITM program of the ESD system were assessed through expert opinion (see Table 2). From the review of studies, ten factors were identified, while an additional three factors were selected based on expert input. The total of thirteen screened and confirmed factors is detailed in Table 2. These factors, identified through the fuzzy Delphi study, include: training, communication, planning, monitoring, standards, instructions and procedures, documentation, physical asset management, hazard identification and risk management, failure management, equipment management and process safety critical tasks, equipment and infrastructure age, the use of new technologies and artificial intelligence, and operational discipline (Fig. 2).

**Table 2:** Results of the first and second-time fuzzy Delphi of the effective factors.

Effective factors	Definite means of the first time	Definite mean of the second time	Difference	Status
Training	0.76	0.78	0.02	Confirmation
Communication	0.72	0.75	0.03	Confirmation
Planning	0.78	0.84	0.06	Confirmation
Monitoring	0.76	0.84	0.08	Confirmation
Standards, guidelines, and procedures	0.75	0.78	0.03	Confirmation
Documentation	0.76	0.78	0.02	Confirmation
Physical asset management	0.75	0.78	0.03	Confirmation
Hazard identification and risk management	0.78	0.84	0.06	Confirmation
Failure management	0.78	0.82	0.04	Confirmation
Equipment management and process safety critical tasks	0.76	0.79	0.03	Confirmation
Equipment and infrastructure age	0.73	0.74	0.01	Confirmation
New technologies and artificial intelligence	0.74	0.76	0.02	Confirmation
Operational discipline	0.82	0.84	0.02	Confirmation

This research presents a conceptual model, illustrated in Fig. 2, that outlines systematic relationships. It offers a comprehensive and consistent description of how individual and organizational factors influence ITM.

After identifying, screening, and validating the factors, we conducted an initial design of the instrument items based on a review of the literature. To assess the face validity of the instrument, we incorporated the revised opinions of experts regarding the formulation of the items and factors. The initial set of 43 questions was then reviewed by experts to confirm the necessity of the items using the content validity ratio index and to evaluate their relevance using the content validity index. Following two stages of evaluation and modification, we finalized a 36-question instrument (see Table 3).

**Table 3:** Content validity ratio (CVR) and content validity index (CVI) of tool items.

Factor	Item	CVR	CVI	Status
ITM	I execute my tasks precisely as planned	0.47	0.8	Reject
	I promptly handle and resolve alarm system notifications	0.87	0.93	Accept
	My tasks are regularly updated to incorporate technological advancements	0.73	0.8	Accept
	I utilize the history of operator actions and recommendations when performing my duties	1	0.93	Accept
	I apply information from equipment manufacturers and vendors in my work	0.73	0.87	Accept
	I perform my tasks with the necessary permissions	0.73	0.93	Accept
Planning	My task routine is based on reliability analysis	0.6	0.8	Accept
	The nature of my tasks is determined by safety considerations and economic factors during planning	0.73	0.8	Accept
	My task plans receive approval from my supervisor	0.33	0.87	Reject
Monitoring	There is a clear procedure for monitoring my tasks	1	0.93	Accept
	I report any overdue or incomplete tasks to my supervisor	0.87	0.87	Accept
	I take timely corrective actions for the system as needed	0.6	0.8	Accept
Standards, guidelines, and procedures	The existing instructions and procedures adequately meet my needs	0.6	0.93	Accept
	The instructions and procedures are clear and practical for implementation	0.73	0.93	Accept
	The instructions are regularly updated	0.87	0.87	Accept
Documentation	I record all completed tasks, either electronically or on paper	0.6	0.87	Accept
Communication	I receive the necessary information to perform my tasks on time	0.6	0.87	Accept
	Communication among my colleagues is effective and clear	0.73	0.73	Accept
	My supervisor provides the information required for my tasks	0.47	0.87	Reject
Training	New colleagues receive comprehensive training to empower them	0.6	0.87	Accept
	The training I receive is sufficient for me to perform my tasks correctly	0.6	0.87	Accept
	Correct task performance is a high priority in the training programs	0.87	0.73	Accept
Physical asset management	The selection and purchase of equipment related to my tasks are conducted efficiently	0.6	0.93	Accept
	The storage of equipment related to my tasks is managed efficiently	0.47	0.87	Reject
	The replacement and disposal of equipment for my tasks are executed efficiently	0.47	0.8	Reject
Hazard identification and risk management	There is an exchange of safety information with risk assessors	0.87	1	Accept
	Awareness of potential hazards associated with my tasks is fostered	0.73	0.8	Accept
	I participate in the root cause determination and analysis of incidents	0.47	0.73	Reject
Failure management	Potential system failures are consistently identified and resolved	0.87	0.87	Accept
	The type and routine of my tasks are based on identified failures and potential failure mechanisms	0.73	1	Accept
	Hidden failures are effectively identified and addressed	0.87	0.87	Accept
Equipment and critical task management	Critical equipment relevant to my tasks is fully identified and regularly updated	1	0.93	Accept
	Special planning is conducted for the inspection, testing, and maintenance of safety–critical elements related to my tasks	0.6	0.93	Accept
	I actively participate in managing safety–critical equipment and processes related to my tasks	0.73	0.93	Accept
Equipment and infrastructure oldness	The age of equipment and infrastructure influences the planning of my tasks	0.87	0.8	Accept
	The age of equipment and infrastructure is effective in monitoring my tasks	1	0.87	Accept
	The age of equipment and infrastructure helps determine the type of tasks I perform	0.33	0.73	Reject
New technologies and artificial intelligence	My company employs modern technologies and artificial intelligence in planning and monitoring	0.87	0.87	Accept
	Modern technologies and artificial intelligence are utilized for inspection, testing, and maintenance	1	0.8	Accept
	These technologies also enhance safety levels within my company	0.6	0.87	Accept
Operational discipline	The start and end times of my tasks are determined optimally	0.87	0.87	Accept
	I perform my tasks at an optimal speed	1	0.87	Accept
	The required manpower and equipment for my tasks are allocated efficiently	0.6	0.87	Accept

The reliability of the instrument was assessed using the split-half method, yielding a coefficient of 0.91, while the Cronbach’s alpha coefficient was found to be 0.97. The internal reliability, which reflects the agreement among evaluators regarding the instrument items, is deemed appropriate. For the pilot study, exploratory factor analysis was conducted using data from 60 participants in the petrochemical industry. The adequacy of the sample was evaluated with the KMO test, yielding a value of 0.75, indicating proximity to one. This suggests that the samples used for factor analysis of the desired variables are sufficiently adequate for this purpose. The Bartlett test indicated a significance level of less than 0.05, confirming the adequacy of the model. From the extracted total variance table, eight factors were identified. These eight latent variables account for 89 percent of the total variance in the manifest variables, with important factors exhibiting eigenvalues greater than one. Notably, only eight variables had total eigenvalues exceeding one, as shown in Table 4.

**Table 4:** Total variance explained.

Component	Initial eigenvalues			Extraction sums of squared loadings			Rotation sums of squared loadings		
	Total	% of Variance	Cumulative %	Total	% of Variance	Cumulative %	Total	% of Variance	Cumulative %
1	16.764	46.565	46.565	16.764	46.565	46.565	4.898	13.605	13.605
2	4.081	11.335	57.900	4.081	11.335	57.900	4.853	13.482	27.087
3	2.715	7.541	65.442	2.715	7.541	65.442	4.817	13.381	40.467
4	2.138	5.940	71.382	2.138	5.940	71.382	3.922	10.894	51.362
5	1.914	5.318	76.700	1.914	5.318	76.700	3.833	10.646	62.008
6	1.835	5.097	81.797	1.835	5.097	81.797	3.378	9.384	71.392
7	1.426	3.961	85.758	1.426	3.961	85.758	3.306	9.183	80.575
8	1.233	3.425	89.184	1.233	3.425	89.184	3.099	8.609	89.184
9	0.589	1.635	90.819						
10	0.446	1.239	92.058						
11	0.389	1.079	93.137						
12	0.302	0.840	93.978						
13	0.290	0.805	94.782						
14	0.256	0.711	95.493						
15	0.234	0.651	96.144						
16	0.224	0.621	96.766						
17	0.159	0.441	97.207						
18	0.153	0.424	97.631						
19	0.130	0.361	97.992						
20	0.117	0.326	98.318						
21	0.108	0.301	98.619						
22	0.095	0.265	98.884						
23	0.091	0.253	99.136						
24	0.068	0.188	99.325						
25	0.052	0.145	99.470						
26	0.043	0.119	99.589						
27	0.033	0.091	99.680						
28	0.030	0.084	99.764						
29	0.024	0.067	99.831						
30	0.017	0.048	99.879						
31	0.014	0.038	99.917						
32	0.011	0.032	99.949						
33	0.008	0.021	99.970						
34	0.005	0.014	99.985						
35	0.004	0.010	99.995						
36	0.002	0.005	100.00						

**Fig. 3:**
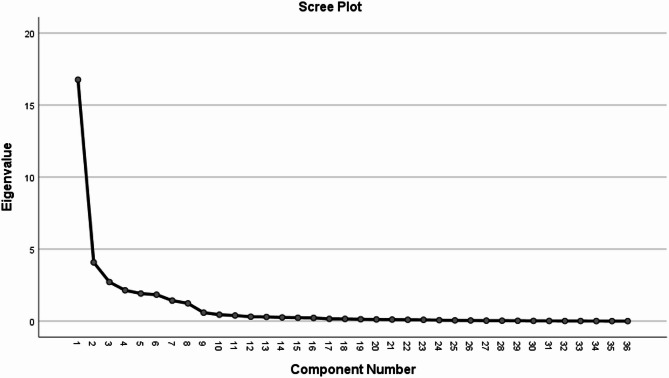
Scree plot.

Figure 3 illustrates the extracted eigenvalues of the explanatory variance for the potential latent variables. Starting from the eighth factor, the figure shows a significant decline in the variance explained by the subsequent variables.

The final clustering of the rotated component matrix has been completed. Table 5 displays the relationships between the manifest variables and the eight extracted latent variables. The main factors were identified, with each item assigned to the factor that exhibited the highest correlation. These factors were named based on the nature of the items, resulting in the following labels: ITM, planning, monitoring, supervision and support, documentation, communication, training, safety, and new technologies and artificial intelligence.

**Table 5:** Rotated Component Matrixa.

	Component							
	1	2	3	4	5	6	7	8
Q1	0.192	**0.898**	0.101	0.189	0.068	0.082	0.170	0.234
Q2	0.156	0.133	0.293	0.167	**0.812**	− 0.044	− 0.020	0.111
Q3	0.144	**0.865**	0.080	0.268	0.096	0.025	0.074	0.173
Q4	0.148	**0.838**	0.171	0.165	0.097	0.010	0.211	0.241
Q5	0.142	**0.902**	0.085	0.118	0.094	0.104	0.124	0.189
Q6	**0.866**	0.236	0.164	0.269	0.128	0.084	0.092	0.068
Q7	**0.858**	0.204	0.179	0.257	0.162	0.065	0.094	0.061
Q8	0.335	0.288	0.187	**0.803**	0.184	0.142	0.091	0.111
Q9	0.315	0.272	0.199	**0.783**	0.200	0.190	0.129	0.160
Q10	0.156	0.271	0.142	0.085	0.197	0.144	0.123	**0.751**
Q11	0.155	0.278	0.133	0.141	0.133	0.047	0.193	**0.848**
Q12	0.103	0.292	0.123	0.142	0.083	0.097	0.164	**0.847**
Q13	0.322	0.345	0.240	0.245	0.176	− 0.098	− 0.111	**0.607**
Q14	0.178	0.232	0.228	0.166	0.135	0.100	**0.772**	0.175
Q15	0.163	0.092	0.231	0.300	0.157	0.130	**0.767**	0.123
Q16	0.271	0.404	0.105	0.221	0.293	0.097	**0.663**	0.019
Q17	0.054	0.032	0.169	0.131	0.271	**0.838**	0.110	0.111
Q18	0.140	0.154	0.268	0.101	0.074	**0.837**	0.064	0.083
Q19	0.102	0.112	0.380	0.313	0.193	**0.664**	0.039	0.076
Q20	0.244	0.252	0.168	**0.775**	0.183	0.272	0.233	0.214
Q21	0.233	0.151	0.145	0.019	0.028	0.063	**0.869**	0.111
Q22	0.146	0.067	0.294	0.262	0.053	**0.754**	0.127	− 0.043
Q23	− 0.021	0.155	**0.859**	0.098	0.165	0.227	0.258	0.131
Q24	0.101	0.119	**0.874**	0.172	0.238	0.161	0.170	0.114
Q25	0.125	0.007	**0.797**	0.070	0.215	0.354	0.193	0.184
Q26	0.100	0.129	**0.900**	0.150	0.209	0.218	0.132	0.143
Q27	**0.895**	0.184	0.052	0.150	0.041	0.128	0.206	0.136
Q28	0.203	0.129	**0.899**	0.148	0.169	0.182	0.026	0.055
Q29	**0.902**	0.154	0.074	0.189	0.129	0.041	0.141	0.144
Q30	0.310	0.276	0.177	**0.791**	0.204	0.230	0.169	0.139
Q31	0.120	0.102	0.200	0.142	**0.900**	0.202	0.115	0.093
Q32	0.106	0.084	0.204	0.140	**0.898**	0.199	0.165	0.114
Q33	0.093	0.062	0.191	0.162	**0.857**	0.222	0.211	0.192
Q34	**0.824**	0.074	0.028	0.121	0.069	0.199	0.314	0.228
Q35	0.262	**0.735**	0.097	0.159	0.048	0.246	0.186	0.279
Q36	0.203	0.170	0.114	**0.634**	0.166	0.372	0.387	0.168

Extraction Method: Principal Component Analysis.

Rotation Method: Varimax with Kaiser Normalization.

a. Rotation converged in 7 iterations.

Significant values are in bold.

**Fig. 4:**
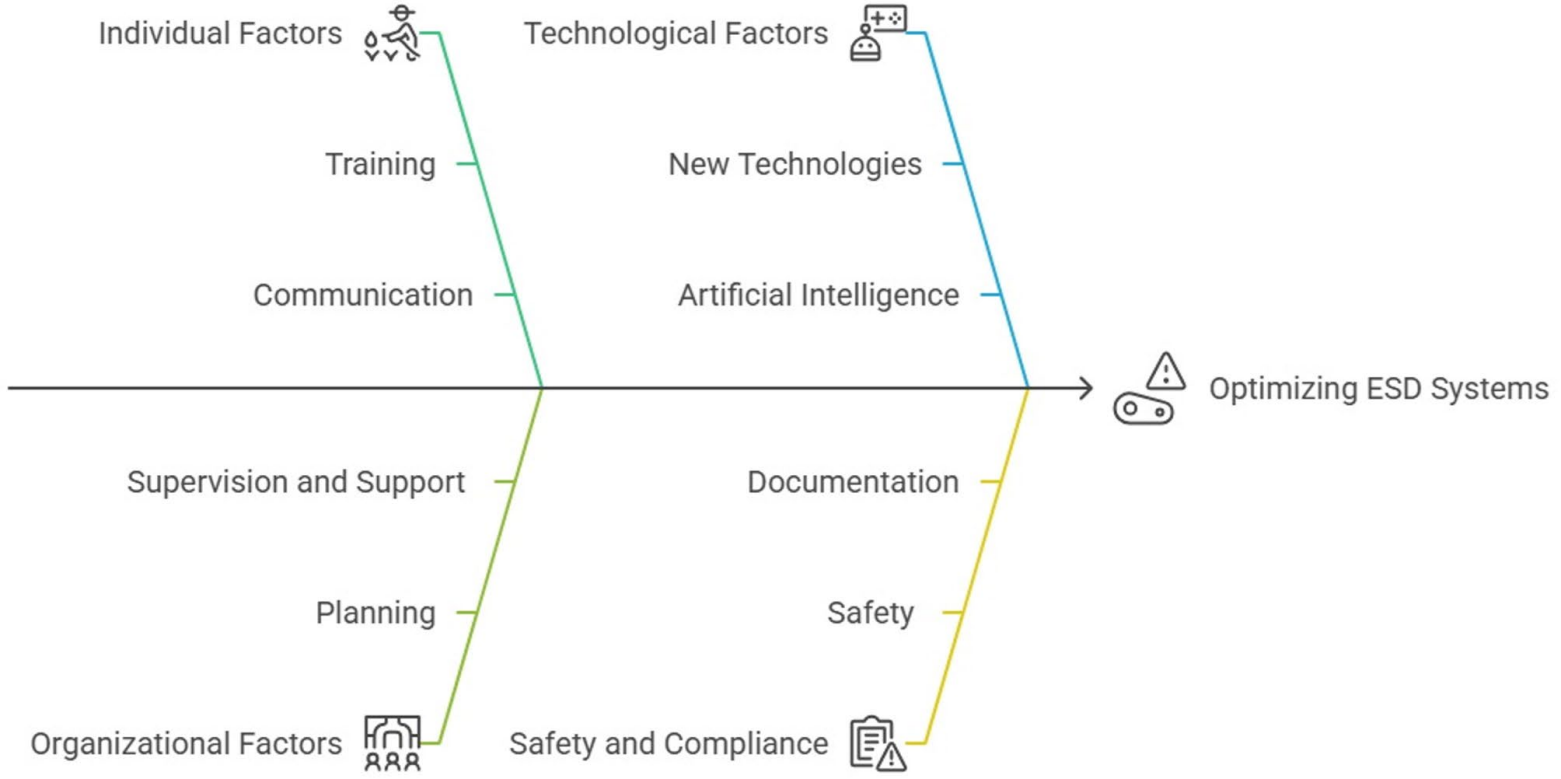
Final factors optimizing the ITM of ESD Systems.

The main structure of the factors and items of the instrument has been formed (Fig. 4, Table 6).

**Table 6:** Factors and items optimizing the ITM of ESD systems.

Factors	Items
ITM	I1. I obtain permission before carrying out my duties
	I2. I promptly address and resolve alarm system notifications
	I3. I utilize the history of operator actions and recommendations in my work
	I4. I incorporate information from equipment manufacturers and vendors in my duties
	I5. I perform my tasks at an optimal speed
Planning	P1. My task routine is organized based on reliability analysis
	P2. The nature of my tasks is determined by safety conditions and economic considerations during planning
	P3. The age of equipment and infrastructure influences my task planning
	P4. Special planning is implemented for the inspection, testing, and maintenance of safety–critical components related to my responsibilities
	P5. The start and end times for my tasks are determined efficiently
Supervision and support	SS1. There is a clear procedure for monitoring my tasks
	SS2. I take timely corrective actions for the system as needed
	SS3. The age of the equipment and infrastructure is considered in monitoring my tasks
	SS4. The selection and acquisition of equipment related to my tasks are conducted efficiently
	SS5. The necessary manpower and equipment are allocated appropriately to my tasks
Documentation	Doc1. I document all tasks I have completed, either electronically or on paper
	Doc2. The instructions and procedures are clear and practical for me to implement
	Doc3. Instructions are regularly updated
	Doc4. The existing instructions and procedures adequately meet all my needs
Communication	Com1. I report any backlogged and uncompleted tasks to my supervisor
	Com2. I receive the information needed to perform my tasks promptly
	Com3. Communication among my colleagues is effective and clear
	Com4. There is an exchange of safety information with risk assessors
Training	Tr1. I am made aware of potential hazards related to my tasks
	Tr2. Ensuring proper task performance is a high priority in our training programs
	Tr3. Comprehensive training is provided to empower new colleagues
	Tr4. The training I receive is sufficient for me to perform my tasks correctly
Safety	S1. I participate actively in managing process safety–critical equipment and tasks
	S2. Critical equipment related to my responsibilities is fully identified and regularly updated
	S3. The type and routine of my tasks are determined based on potential failures and failure mechanisms
	S4. Potential system failures are consistently identified and resolved
	S5. Hidden failures are effectively identified and addressed
Technologies and artificial intelligence	Tech1. My tasks are updated to reflect technological advancements
	Tech2. My company employs new technologies and artificial intelligence to enhance safety and reduce risk
	Tech3. The company is engaged in planning and monitoring new technologies and artificial intelligence
	Tech4. My company utilizes new technologies and artificial intelligence for inspection, testing, and maintenance

**Fig. 5:**
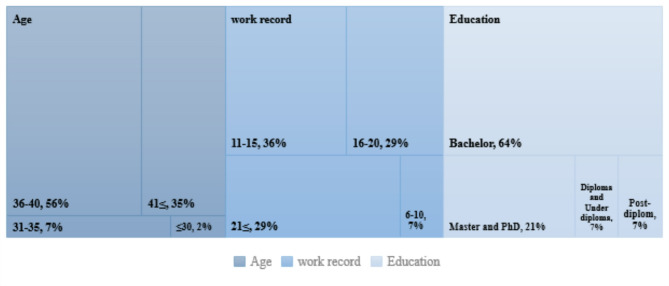
Demographic information.

The second pilot study included a statistical population of 84 participants. Figure 5 presents data on the participants’ age, work record, and education.

Table 7 presents the factor loadings for each research measure used to assess reliability and construct validity. As shown in Table 7, all variable factor loadings exceed 0.7, confirming the reliability and construct validity of the research model measures. The measurement model evaluation included both Cronbach’s alpha coefficient and CR coefficient. All constructs had values for these indices above 0.7, indicating acceptable reliability of the model. Additionally, the AVE for all constructs was above 0.7, demonstrating adequate convergent validity.

**Table 7:** Measurement model.

Factor	Item	Outer loadings	Construct reliability and validity			
			Cronbach’s alpha	Composite reliability (rho_a)	Composite reliability (rho_c)	Average variance extracted (AVE)
ITM	I1	0.935	0.924	0.926	0.943	0.770
	I2	0.861				
	I3	0.920				
	I4	0.868				
	I5	0.797				
Plan	P1	0.938	0.964	0.965	0.972	0.873
	P2	0.933				
	P3	0.949				
	P4	0.928				
	P5	0.923				
Supervision and support	SS1	0.967	0.981	0.981	0.985	0.928
	SS2	0.969				
	SS3	0.966				
	SS4	0.957				
	SS5	0.958				
Safety	Sa1	0.893	0.939	0.945	0.953	0.804
	Sa2	0.908				
	Sa3	0.908				
	Sa4	0.901				
	Sa5	0.872				
Technologies and artificial intelligence	Tech1	0.866	0.947	0.953	0.962	0.864
	Tech2	0.952				
	Tech3	0.962				
	Tech4	0.935				
Training	Tr1	0.908	0.881	0.883	0.920	0.742
	Tr2	0.904				
	Tr3	0.885				
	Tr4	0.737				
Communication	Com1	0.957	0.956	0.961	0.969	0.885
	Com2	0.966				
	Com3	0.964				
	Com4	0.874				
Documentation	Doc1	0.951	0.880	0.960	0.909	0.717
	Doc2	0.922				
	Doc3	0.711				
	Doc4	0.781				

**Fig. 6:**
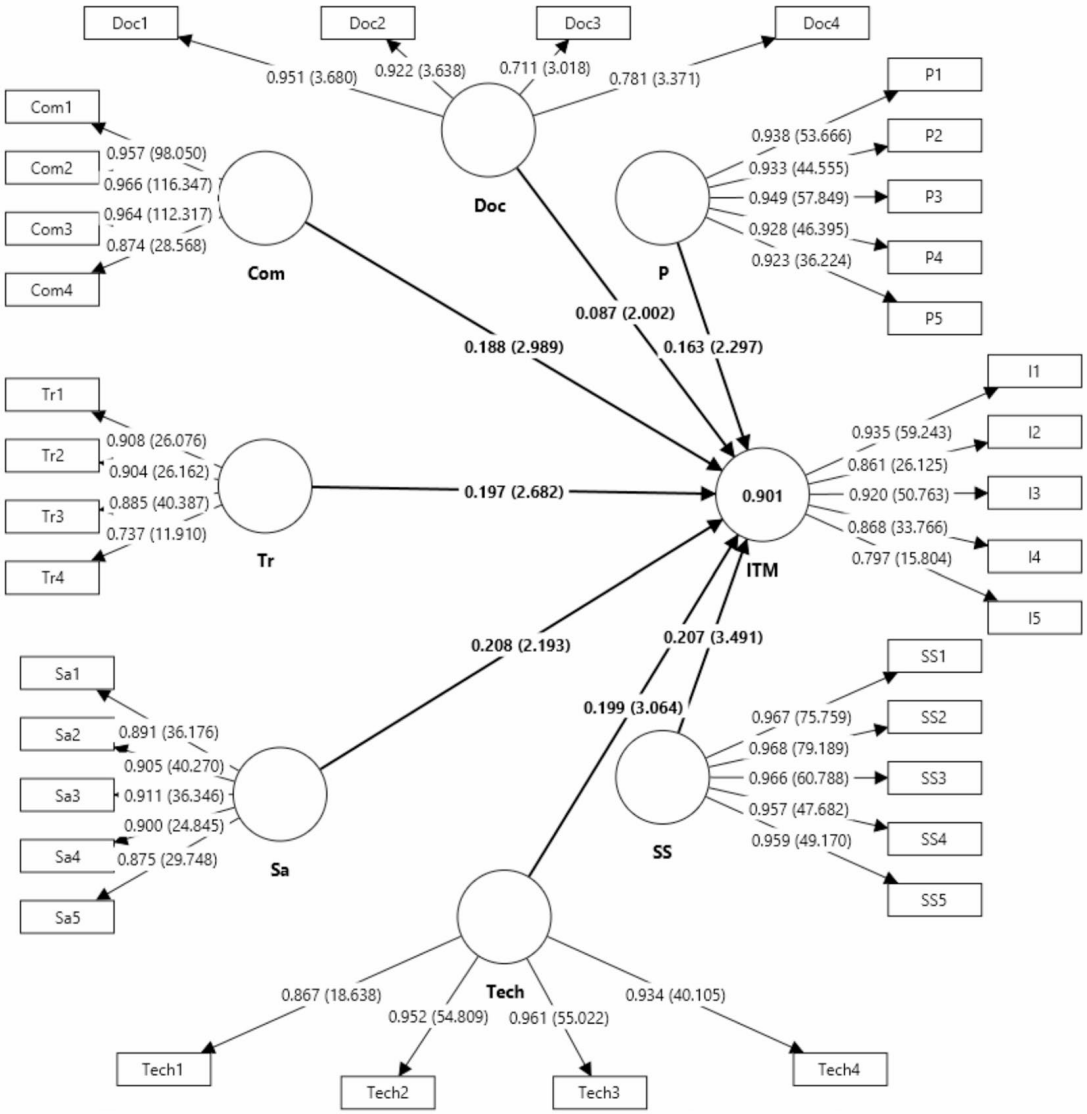
Structural equation modeling.

Table 8 presents results indicating that, according to the Fornell & Larcker criterion, the values in the main diagonal of the matrix (representing each construct) are greater than the correlation values between constructs (represented by the values outside the diagonal). This confirms that each construct is sufficiently differentiated from the others in the model. Additionally, the cross-loadings factor is shown in Table 8, revealing that each index has the highest factor loading on its construct, while the factor loadings on other constructs are significantly lower. These findings further support the appropriate differentiation between constructs. The values of the HTMT index for all pairs of constructs were calculated and compared against a threshold of 0.9, and in some cases, 0.85. The results from Table 8 indicate that all HTMT index values fall below this threshold. Lastly, the goodness-of-fit criteria for the research model were evaluated, with the structural model reporting an SRMR criterion of 0.071. This result suggests that the model demonstrates a good fit (Fig. 6).

**Table 8:** Discriminant validity.

Fornell &Larcker criterion	Com	Doc	ITM	P	SS	Sa	Tech	Tr
Com	0.941							
Doc	− 0.222	0.847						
ITM	0.814	− 0.125	0.877					
P	0.672	− 0.150	0.799	0.934				
SS	0.637	− 0.226	0.733	0.543	0.963			
Sa	0.738	− 0.118	0.839	0.737	0.552	0.897		
Tech	0.612	− 0.300	0.721	0.554	0.573	0.598	0.929	
Tr	0.651	− 0.074	0.806	0.743	0.552	0.756	0.503	0.862
**Cross loadings**	Com	Doc	ITM	P	SS	Sa	Tech	Tr
Com1	**0.957**	− 0.274	0.795	0.632	0.628	0.696	0.605	0.602
Com2	**0.966**	− 0.224	0.790	0.649	0.628	0.699	0.616	0.643
Com3	**0.964**	− 0.230	0.789	0.673	0.597	0.726	0.638	0.632
Com4	**0.874**	− 0.095	0.683	0.569	0.540	0.654	0.426	0.573
Doc1	− 0.222	**0.951**	− 0.142	− 0.167	− 0.262	− 0.143	− 0.314	− 0.074
Doc2	− 0.207	**0.922**	− 0.116	− 0.135	− 0.246	− 0.082	− 0.296	− 0.049
Doc3	− 0.148	**0.711**	− 0.015	− 0.097	− 0.110	0.042	− 0.075	− 0.055
Doc4	− 0.150	**0.781**	− 0.064	− 0.071	− 0.018	− 0.100	− 0.168	− 0.084
I1	0.761	− 0.079	**0.935**	0.706	0.643	0.782	0.653	0.773
I2	0.694	0.079	**0.861**	0.625	0.495	0.695	0.520	0.749
I3	0.669	0.001	**0.920**	0.750	0.604	0.754	0.624	0.725
I4	0.732	− 0.271	**0.868**	0.758	0.653	0.759	0.726	0.680
I5	0.707	− 0.256	**0.797**	0.653	0.808	0.684	0.623	0.609
P1	0.625	− 0.108	0.754	**0.938**	0.477	0.683	0.499	0.729
P2	0.605	− 0.130	0.748	**0.933**	0.524	0.659	0.540	0.714
P3	0.632	− 0.155	0.780	**0.949**	0.558	0.680	0.500	0.677
P4	0.609	− 0.177	0.689	**0.928**	0.479	0.700	0.500	0.623
P5	0.664	− 0.134	0.754	**0.923**	0.495	0.723	0.547	0.722
SS1	0.603	− 0.210	0.671	0.464	**0.967**	0.491	0.546	0.472
SS2	0.649	− 0.266	0.724	0.553	**0.968**	0.564	0.580	0.553
SS3	0.616	− 0.248	0.699	0.535	**0.966**	0.521	0.557	0.545
SS4	0.596	− 0.160	0.708	0.498	**0.957**	0.529	0.537	0.519
SS5	0.602	− 0.206	0.727	0.559	**0.959**	0.548	0.541	0.564
Sa1	0.716	− 0.165	0.780	0.654	0.535	**0.891**	0.569	0.642
Sa2	0.749	− 0.156	0.833	0.686	0.514	**0.905**	0.651	0.715
Sa3	0.618	− 0.062	0.711	0.687	0.508	**0.911**	0.456	0.646
Sa4	0.602	− 0.110	0.714	0.619	0.462	**0.900**	0.535	0.706
Sa5	0.602	− 0.021	0.709	0.654	0.448	**0.875**	0.448	0.680
Tech1	0.546	− 0.329	0.600	0.374	0.487	0.466	**0.867**	0.396
Tech2	0.594	− 0.264	0.726	0.577	0.578	0.606	**0.952**	0.472
Tech3	0.556	− 0.289	0.680	0.553	0.535	0.572	**0.961**	0.472
Tech4	0.576	− 0.240	0.665	0.537	0.526	0.571	**0.934**	0.527
Tr1	0.485	− 0.096	0.677	0.689	0.458	0.633	0.378	**0.908**
Tr2	0.533	− 0.078	0.609	0.638	0.398	0.609	0.340	**0.904**
Tr3	0.665	− 0.226	0.754	0.700	0.585	0.687	0.550	**0.885**
Tr4	0.537	0.155	0.707	0.517	0.431	0.656	0.434	**0.737**
**Heterotrait-monotrait ratio (HTMT)**	Com	Doc	ITM	P	SS	Sa	Tech	Tr
Com								
Doc	0.227							
ITM	0.864	0.157						
P	0.698	0.149	0.843					
SS	0.657	0.204	0.767	0.556				
Sa	0.774	0.124	0.896	0.774	0.572			
Tech	0.639	0.275	0.766	0.575	0.594	0.627		
Tr	0.704	0.192	0.887	0.803	0.585	0.826	0.541	

Significant values are in bold.

## Discussion

**Fig. 7:**
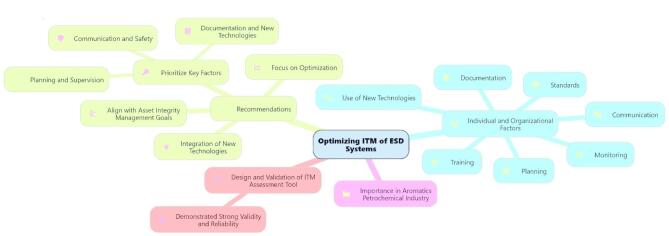
Optimization of the ITM of ESD systems mind map.

Optimizing the ITM of ESD systems in the petrochemical industry is vital for ensuring operational safety and minimizing risks associated with hazardous processes. This study aims to investigate the individual and organizational factors influencing the effectiveness of ITM practices and to develop a reliable tool for assessing these factors within the AIM framework. Key factors affecting ITM include planning, supervision and support, documentation, communication, training, safety, new technologies, and artificial intelligence. The findings highlight that effective planning is crucial for the successful implementation of ITM. Sheikhalishahi et al. reviewed an integrated approach to maintenance planning that considers human factors in a petrochemical plant. A new maintenance planning approach, which incorporates grouping strategies and human factors, is presented. This approach outlines various steps from system configuration to the review of maintenance plans. Continuous training and professional development are essential for enhancing personnel competencies. The dynamic nature of the petrochemical industry necessitates ongoing training to keep personnel updated with the latest technologies and best practices^66^. Al-Daghan et al. examined the roles of knowledge, training, and skills in boosting employee productivity, as well as the mediating role of employee safety. The current study investigates the impact of employee safety knowledge, safety training, and safety skills on overall employee safety. The target population includes employees of petrochemical companies in Saudi Arabia. Results indicate that employee safety knowledge, training, and skills significantly influence safety outcomes. Moreover, employee safety positively impacts productivity. This study recommends that managers seeking to enhance employee productivity should prioritize safety training, safety knowledge, safety skills, and employee safety awareness^67^. Dasgupta et al. conducted a systematic review of engineering management perspectives on safety culture in chemical and petrochemical plants. The results reveal that safety performance in these industries is closely linked to active management engagement in promoting safety practices, effective communication strategies, and ongoing evaluation and enhancement of safety protocols. This review offers valuable insights and practical guidelines for engineering managers aiming to strengthen safety protocols, mitigate risks, and improve safety performance in high-risk industrial environments^68^. Hakim et al. analyzed safety communication within the industry. Effective safety communication is essential for improving safe and healthy working conditions by emphasizing the identification and mitigation of workplace hazards. Safety conversations are pivotal in preventing workplace accidents and enhancing workers’ understanding of occupational safety and health. Safety conversations and communication focus on enhancing safety culture and workplace performance^69^. Supervision and support are critical factors that contribute to the success of optimizing ITM processes. Prioritizing asset integrity and allocating proper resources to ITM activities are essential for effective safety management. New technologies and artificial intelligence play a significant role in enhancing the overall effectiveness of maintenance programs. The interplay between supervision, support, and new technologies can lead to improved safety outcomes and operational reliability. Yousef et al. examined the influence of technology management on sustainability performance in Egyptian oil refineries and petrochemical companies, finding that it has a direct and significant impact on sustainability. The study highlights that process technology positively affects three key aspects of sustainability. However, while maintenance technology improves economic and environmental sustainability, it does not directly impact social sustainability^6^. He et al. introduced a real-time probabilistic risk assessment method for the petrochemical industry that utilizes data monitoring. This research advances the field by proposing a risk update method based on a dynamic Bayesian network, integrating data monitoring into real-time assessments^70^. Zhi-Hai et al. investigated the use of image monitoring, detection technology, and intelligent inspection robots in the petrochemical industry, where the handling of flammable and explosive materials makes timely detection of abnormalities crucial for production safety. This paper outlines an approach to building a digital twin system for petrochemical processes, proposing a detailed logical structure for monitoring chemical process status and diagnosing faults, thereby enhancing safety and controllability^71^. Li et al. reviewed the exploration and application of intelligent monitoring and analysis technologies in the petrochemical industry. With the rapid advancement of computer vision, pattern recognition, and other technologies, petrochemical enterprises are increasingly demanding superior intelligent monitoring capabilities, leading to continuous detection and analysis of activities within various visual contexts. The increasing emphasis on continuous detection and control in the production areas of petrochemical enterprises supports the realization of intelligent management. Integrating ITM practices into the AIM framework fosters a more systematic approach to managing asset risk and performance^72^. This study highlights the importance of adopting a comprehensive perspective that includes technical, managerial, and operational considerations. By implementing the AIM approach, organizations can better align their ITM activities with business objectives, optimize resource allocation, and enhance overall system reliability. The design and validation of the tool were crucial steps in systematically evaluating the factors influencing ITM practices. A rigorous validation process ensured that the instrument was reliable and effectively captured the complexities of individual and organizational influences within the petrochemical context. The implications of these findings are diverse. Organizations should invest in appropriate training programs tailored to the specific needs of their workforce. Using a validated instrument as a benchmark can help identify areas for improvement and guide strategic initiatives related to asset integrity. Examining improvements in planning, supervision and support, documentation, communication, training, safety, and the integration of new technologies and artificial intelligence in optimizing ESD systems can yield better outcomes. The optimization of the ITM of ESD systems provided the mind map in Fig. 7.

This study has several limitations. It was conducted as a cross-sectional study, and one limitation of cross-sectional studies is their inability to establish causality. Cross-sectional studies are also prone to selection bias and the presence of confounding variables. To mitigate these effects as much as possible, multi-source methods (literature review and expert opinions) were used, but residual biases may still impact the results. The sampling was limited to a few petrochemical complexes with specific organizational structures and maintenance policies. Therefore, the findings may not fully represent all types of petrochemical units or other process industries. This may reduce the generalizability of the results to other countries or sectors, such as oil and gas, refineries, or chemical plants. Factors such as political, economic, and social changes in countries may also affect the study’s outcomes. The selection of suitable participants to validate individual and organizational factors may have its own limitations. Although participants had over five years of specialized experience, the relatively small and homogeneous expert sample may have introduced selection bias and limited the diversity of perspectives. Data collection was based on self-reported questionnaires conducted both in person and online. Self-reported measures are susceptible to recall bias and response inaccuracies, which can affect the results. Responses to questionnaires may also be influenced by the design of the questions and the respondents’ interpretations, particularly in specific cultural contexts where individuals may be reluctant to express their issues.

For future studies, several recommendations are proposed. Developing and validating tools across international samples and diverse industries, as well as conducting longitudinal studies, is suggested. Longitudinal studies would help identify causal relationships, reduce bias, and pinpoint influential factors. They could provide deeper insights into how individual and organizational factors affect the effectiveness of inspection, testing, and preventive maintenance over time. Including a larger and more geographically diverse expert panel could enhance the generalizability of the findings. Additionally, the tools designed to assess the effectiveness of inspection, testing, and maintenance programs require further validation in environments and countries with different maintenance models.

## Conclusion

To optimize the Inspection, Testing, and Maintenance (ITM) of Emergency Shutdown (ESD) systems, which are critical for enhancing safety in the petrochemical industry, a robust Asset Integrity Management (AIM) system is essential. The research focused on two main aspects: identifying individual and organizational factors that influence the optimization of these systems and developing and validating a tool for comprehensive and accurate data collection and assessment. The study identified several key individual and organizational factors, including training, communication, planning, monitoring, standards, guidelines, procedures, documentation, physical asset management, hazard identification and risk management, failure management, equipment management, critical process safety tasks, equipment and infrastructure age, adoption of new technologies and artificial intelligence, and operational discipline. Addressing these factors not only improves the efficiency of ITM for ESD systems but also aligns with the broader objectives of AIM in the petrochemical industry. The tool designed and validated for ITM of ESD systems was found to be highly reliable and valid. Its development and testing underscored the importance of precise tools for evaluating the current state of ESD systems and their future requirements. These tools can help the petrochemical industry review and enhance its regulations and policies. The study’s findings indicate that an integrated approach to asset management, combined with improvements in individual and organizational factors, can significantly improve ESD systems in the petrochemical industry. This study provides a comprehensive, empirically validated theory and measurement tool for optimizing ITM of ESD systems. The model integrates human, organizational, and technological factors to create a pathway for enhancing safety, reliability, and resilience in critical process control systems.

Three practical applications emerge from this study. First, optimizing ITM requires integrated interventions that combine effective planning and monitoring with focused training, transparent documentation, and regular oversight. Second, for new technologies and artificial intelligence to contribute to safety, there must be established new technologies and artificial intelligence to help procedures, training, and contingency planning for failures. Third, operators and safety managers can periodically use this tool to identify gaps, set improvement priorities, and track continuous progress over time.

## Data Availability

The datasets generated during and/or analysed during the current study are not publicly available but are available from the corresponding author on reasonable request.
